# Supplementary material for: Release factor-dependent ribosome rescue by BrfA in the Gram-positive bacterium Bacillus subtilis
Source: Nat Commun. 2019 Nov 27;10:5397. doi: 10.1038/s41467-019-13408-7 (PMC6881298; doi:10.1038/s41467-019-13408-7)
Supplement: Supplementary file 1 — Summlementary Information [file 41467_2019_13408_MOESM1_ESM.pdf]

Supplementary Information

Release factor-dependent ribosome rescue by BrfA in the Gram-positive bacterium *Bacillus subtilis*

Shimokawa-Chiba et al.

Supplementary Tables

Supplementary Table 1. Data collection, refinement and validation statistics

	**BrfA-RF2-ns70S**	
**Data collection**		
Particles	154,405	
Microscope	Titan Krios	
Detector	Falcon III	
Pixel size (Ǻ)	1.065	
Defocus range (μm)	0.4-2.2	
Voltage (keV)	300	
Electron dose (e^-/Ǻ^2)		2.0
**Model composition**		
Protein residues	6100	
RNA nucleotides	4646	
Hydrogens	0	
**Refinement**		
Resolution (Ǻ)		3.06
Map sharpening B factor (Ǻ^2)	-73.0479	
CC map/model	0.85	
**Validation: proteins**		
Poor rotamers (%)	0.54	
Ramachandran outliers (%)		0.22
Bad backbone bonds (%)	0.01	
Bad backbone angels (%)	0.05	
**Validation: RNA**		
Correct sugar puckers (%)		99.31
Good backbone conformations (%)	79.88	
Bad bonds (%)	0.00	
Bad angels (%)	0.02	
**Scores**		
MolProbity		1.74 (88th percentile)
Clash score, all atoms		4.98 (93nd percentile)

Supplementary Table 2. *E. coli* strains

Name	Description	Reference
BL21(DE3)	F^-, *ompT*, *hsdS*$_B$(r_B^- m_B^-), *gal*(*λcI 857, ind1, Sam7, nin5, lacUV5-T7gene1*), *dcm*(DE3)	Promega
NAE970	BL21(DE3)/pNAR913 (*Bs_prfA-his*$_6$)	This study
NAE972	BL21(DE3)/pNAR915 (*Bs_prfB-his*$_6$)	This study
NAE973	BL21(DE3)/pNAR916 (*brfA62-his*$_6$)	This study
NAE982	BL21(DE3)/pNAR917 (*his*$_6$*-arfA(2-60)*)	This study
NAE1003	BL21(DE3)/pCH2307 (*Bs_prfB(GAQ)-his*$_6$)	This study
NAE1017	BL21(DE3)/pNAR939 (*Bs_pfrB(SPT)-his*$_6$)	This study

Supplementary Table 3. *B. subtilis* strains and construction

Name	Description	Reference	Construction	
			Host	DNA
PY79	*WT*	ref. [1]		
BKE23540	*ΔbrfA::erm*	ref. [2]		
BKE33600	*ΔsmpB::erm*	ref. [2]		
D1	*ΔssrA::cat*	ref. [3]		
TSB2	*ΔssrA::cat::tet*	This study	SCB2582	pCm::Tc
NAB1196	*thrC::*P_{xylA} *GFPΩerm*	This study	PY79	pNAR778
NAB1198	*thrC::*P_{xylA} *GFP-nsΩerm*	This study	PY79	pNAR780
NAB1200	*ΔssrA::cat, thrC::*P_{xylA} *GFPΩerm*	This study	SCB2582	pNAR778
NAB1202	*ΔssrA::cat, thrC::*P_{xylA} *GFP-nsΩerm*	This study	SCB2582	pNAR780
NAB1280	*ΔsmpB::erm*	This study	PY79	BKE33600
NAB1281	*ΔyesZ::loxP, ΔlacA::loxP, ΔsmpB::erm*	This study	KFB793	NAB1233
NAB1282	*ΔsmpB::loxP*	This study	NAB1280	pMK2
NAB1283	*ΔyesZ::loxP, ΔlacA::loxP, ΔsmpB::loxP*	This study	NAB1281	pMK2
NAB1286	*ΔyesZ::loxP, ΔlacA::loxP, ΔsmpB::loxP / pNAR813*	This study	NAB1283	pNAR813
NAB1298	*ΔbrfA::erm*	This study	PY79	BKE23540
NAB1346	*ΔbrfA::kan*	This study	PY79	pNAR903
NAB1509	*amyE::*P_{mifM} *rbsm1-GFP-brfA-FLAGΩlacZΩcat*	This study	PY79	pNAR1036
NAB1510	*amyE::*P_{mifM} *rbsm1-GFP-brfA62-FLAGΩlacZΩcat*	This study	PY79	pNAR1037
NAB1511	*amyE::*P_{mifM} *rbsm1-GFP-brfA(no_term)-FLAGΩlacZΩcat*	This study	PY79	pNAR1038
NAB1514	*ΔsmpB::erm, amyE::*P_{mifM} *rbsm1-GFP-brfA-FLAGΩlacZΩcat*	This study	NAB1280	pNAR1036
NAB1515	*ΔsmpB::erm,,amyE::*P_{mifM} *rbsm1-GFP-brfA62-FLAGΩlacZΩcat*	This study	NAB1280	pNAR1037
NAB1516	*ΔsmpB::erm,,amyE::*P_{mifM} *rbsm1-GFP-brfA(no_term)-FLAGΩlacZΩcat*	This study	NAB1280	pNAR1038
SCB851	*amyE::*P_{mifM} *rbsm1-GFP-mifM35-flag-yidC2'-lacZΩcat*	ref. [4]		
SCB2582	*ΔssrA::cat*	This study	PY79	D1
SCB4122	*amyE::*P_{mifM} *rbsm1 GFP-brfA62-FLAGΩcat*	This study	PY79	pCH2240
SCB4126	*ΔsmpB::erm, amyE::*P_{mifM} *rbsm1 GFP-brfA62-FLAGΩcat*	This study	NAB1280	pCH2240
SCB4141	*amyE::*P_{mifM} *rbsm1-GFP-brfA-FLAGΩcat*	This study	PY79	pCH2239
SCB4142	*amyE::*P_{mifM} *rbsm1-GFP-brfA(no_term)-FLAGΩcat*	This study	PY79	pCH2241
SCB4144	*ΔsmpB::erm, amyE::*P_{mifM} *rbsm1-GFP-brfA-FLAGΩcat*	This study	NAB1280	pCH2239

SCB4145	*ΔsmpB::erm, amyE::*P_{mifM} *rbsm1-GFP-brfA(no_term)-FLAGΩcat*	This study	NAB1280	pCH2241
SCB4153	*lacA::*P_{xylA} *dCas9Ωerm, amyE::*P_{veg} *sgRNA-smpBΩcat*	This study	KFB946	pCH2264
SCB4191	*lacA::*P_{xylA} *dCas9Ωerm, ΔbrfA::kanR*	This study	KFB946	NAB1346
SCB4194	*amyE::*P_{veg} *sgRNA-smpBΩcat, lacA::*P_{xylA} *dCas9Ωerm, ΔbrfA::kanR*	This study	SCB4191	pCH2264
SCB4199	*amyE::*P_{veg} *sgRNA-ssrAΩcat, lacA::*P_{xylA} *dCas9Ωerm, ΔbrfA::kanR*	This study	SCB4191	pCH2302
SCB4205	*lacA::*P_{xylA} *dCas9Ωerm, amyE::*P_{veg} *sgRNA-ssrAΩcat*	This study	KFB946	pCH2302
SCB4215	*amyE::*P_{veg} *sgRNA-smpBΩcat, lacA::*P_{xylA} *dCas9Ωerm, ΔbrfA::kanR, thrC::PbrfA brfAΩspc*	This study	SCB4194	pCH2293
SCB4217	*amyE::*P_{veg} *sgRNA-ssrAΩcat, lacA::*P_{xylA} *dCas9Ωerm, ΔbrfA::kanR, thrC::PbrfA brfAΩspc*	This study	SCB4199	pCH2293
SCB4218	*amyE::*P_{mifM} *rbsm1-GFP-mifM35-flag-yidC2'-lacZΩcat, ssrA::cat::tet*	This study	SCB851	TSB2
KFB792	*ΔlacA::loxP-kanR*	This study	PY79	DNA fragment
KFB793	*ΔyesZ::loxP, ΔlacA::loxP*	This study	KYB112	KFB792, pMK2
KFB946	*lacA::*P_{xylA} *dCas9Ωerm*	This study	PY79	pJMP1
KFB948	*ΔyesZ::loxP, lacA::Pxyl-dCas9Ωerm*	This study	KYB112	pJMP1
KYB112	*ΔyesZ::loxP*	This study	PY79	pKY13, pMK2

Supplementary Table 4. Plasmids

Name	Description	Source
pCm::Tc	*Cm::Tc*	ref. [5]
pDG1664	*thrC::erm* integration vector	ref. [6]
pET28b	vector	Novagen
pLOSS*	Ts vector	ref. [7]
pyqjG21	*amyE::*P_{mifM} *mifM-yidC2-gfp*	ref. [4]
pJMP1	*lacA::Pxyl dCas9Ωerm*	ref. [8]
pNAR756	P_{xylA} *yidC2*	This study
pNAR758	P_{xylA} *yidC2-non_stop*	This study
pNAR778	*thrC::*P_{xylA} *GFPΩerm*	This study
pNAR780	*thrC::*P_{xylA} *GFP-nsΩerm*	This study
pNAR809	P_{spac} *smpB*	This study
pNAR813	P_{spac} *smpB-FLAG*	This study
pNAR869	*brfA-*his_6	This study
pNAR879	*amyE::*P_{mifM} *rbsm1-GFP-brfAΩcat*	This study
pNAR901	upstream region of *brfA*	This study
pNAR903	*ΔbrfA::kan*	This study
pNAR913	*Bs_prfA-*his_6	This study
pNAR915	*Bs_prfB-*his_6	This study

pNAR916	*brfA62-*his_6	This study
pNAR917	his_6*-arfA(2-60)*	This study
pNAR939	*Bs_pfrB(SPT)*	This study
pNAR1036	*amyE::*P_{mifM} *rbsm1-GFP-brfA-FLAGΩlacZΩcat*	This study
pNAR1037	*amyE::*P_{mifM} *rbsm1-GFP-brfA62-FLAGΩlacZΩcat*	This study
pNAR1038	*amyE::*P_{mifM} *rbsm1-GFP-brfA(no_term)-FLAGΩlacZΩcat*	This study
pCH735	*amyE::*P_{mifM} *mifM-lacZΩcat*	ref. [4]
pCH747	*amyE::*P_{mifM} *mifM-gfpΩcat*	This study
pCH805	*amyE::*P_{mifM} *GFP-mifM35-yidC2'-lacZΩcat*	ref. [4]
pCH913	*amyE::*P_{mifM} *rbsm1-GFP-mifM35-yidC2'-lacZΩcat*	ref. [4]
pCH1141	*thrC::*P_{xylA}*Ωerm*	ref. [9]
pCH1142	*kanΩspc*	ref. [10]
pCH1517	*amyE::PyqzJ rbsm1-GFP-mifM35-95-lacZΩcat*	ref. [10]
pCH2238	his_6*-Bs_prfB*	This study
pCH2239	*amyE::*P_{mifM} *rbsm1 GFP-brfA-FLAGΩcat*	This study
pCH2240	*amyE::*P_{mifM} *rbsm1 GFP-brfA62-FLAGΩcat*	This study
pCH2241	*amyE::*P_{mifM} *rbsm1 GFP-brfA(no_term)-FLAGΩcat*	This study
pCH2264	*amyE::*P_{veg} *sgRNA-smpBΩcat*	This study
pCH2293	*thrC::*P_{brfA} *brfAΩspc*	This study
pCH2302	*amyE::*P_{veg} *sgRNA-ssrAΩcat*	This study
pCH2307	*Bs_prfB(GAQ)-*his_6	This study
pKIG855	*amyE::*P_{veg} *sgRNA-rfpΩcat*	This study
pMK2	*Pspac cre*	This study
pKY13	*ΔyesZ::loxP-kan-loxP*	This study
pNR1	*amyE::*P_{xylA} *rbsm1 mifM-GFPΩcat*	This study

Supplementary Table 5. Primers

Name	Sequence
SP1	TATTTTTAAGGGGGAAATCACATAAAAAGGAGGAGAACAAAA
SP2	GAAGCTTATCGAATTCACTAGTTCAGCCATGATAAACAAGACTG
SP3	AGTCTTGTTTATCATGGCTGAACTAGTGAATTCGATAAGCTTC
SP4	TTTTGTTCTCCTCCTTTTTATGTGATTTCCCCCTTAAAAATA
SP5	GTGAAAAATAAAAGCAACCCCGTGCAAAAAG
SP6	GGGGTTGCTTTTATTTTTCACCGACTCAGTAAGAGC
SP7	GAACAAAATTGTTAAAAACATATGGATCCAAGCTTACTAGTAGT
SP8	GAAGCTTATCGAATTCACTAGTTCACGGCCGTTTGTATAGTTCATC

SP9 GATGAACTATACAAACGGCCGTGAACTAGTGAATTCGATAAGCTTC

SP10 ACTACTAGTAAGCTTGGATCCATATGTTTTAACAATTTTGTTC

SP11 CTTTTTGCACGGGGTTGCTTTTATTCGGCCGTTTGTATAGTTCATC

SP12 GATGAACTATACAAACGGCCGAATAAAAGCAACCCCGTGCAAAAAG

SP13 AGGCCGCGGATGCATAGGCCTTTAGAGAGAGGAGGTTCTGGC

SP14 TGGGGATCCGCATGCACTAGTTTAGAAGCCTTTTTGACTGTC

SP15 GACAGTCAAAAAGGCTTCTAAACTAGTGCATGCGGATCCCCA

SP16 GCCAGAACCTCCTCTCTCTAAAGGCCTATGCATCCGCGGCCT

SP17 CTACAAAGACGATGACGACAAGTAAACTAGTGCATGCGGATCCC

SP18 GTCGTCATCGTCTTTGTAGTCGAAGCCTTTTTGACTGTCTCT

SP19 CTTTAAGAAGGAGATATACCATGGCAAAAAGCCAAGCGAAAAAG

SP20 CTCAGTGGTGGTGGTGGTGGTGGGCGGCTTTTTGGGGCACAAAAAAATC

SP21 GATTTTTTTGTGCCCCAAAAAGCCGCCCACCACCACCACCACCACTGAG

SP22 CTTTTTCGCTTGGCTTTTTGCCATGGTATATCTCCTTCTTAAAG

SP23 CCGCGGGGTGCAACAGGATCCGCAAAAAGCCAAGCGAAAAAG

SP24 CTGGTCTGATCGGATCTCTAGGCGGCTTTTTGGGGCACAAAAAAATC

SP25 GATTTTTTTGTGCCCCAAAAAGCCGCCTAGAGATCCGATCAGACCAG

SP26 CTTTTTCGCTTGGCTTTTTGCGGATCCTGTTGCACCCCGCGG

SP27 GATTATACCGAGGTATGAAAACCTTGGTCTGATAATGGGATTTAC

SP28 TCTGTAAAGGTCCAATTCTCGGTGATCACTCCCTTTTTTATTTTC

SP29 CGAGAATTGGACCTTTACAGA

SP30 TTTTCATACCTCGGTATAATC

SP31 TTACTGGATGAATTGTTTTAGCATTCATCTCTATTGTTTCTT

SP32 TTAGACATCTAAATCTAGGTAGTTTTATACATAGAAACAGCA

SP33 TACCTAGATTTAGATGTCTAAAAAGC

SP34 CTAAAACAATTCATCCAGTAA

SP35 TAACTTTAAGAAGGAGATATACCAATGTTAGACCGTTTAAAATCAA

SP36 TTATTAGTGGTGGTGGTGGTGGTGACCTTCCGACTGCTGAAGCTTG

SP37 CACCACCACCACCACCACTAATAATGAGATCCGGCTGCTAACAAAG

SP38 TGGTATATCTCCTTCTTAAAGTTA

SP39 TAACTTTAAGAAGGAGATATACCAATGGAATTATCAGAAATTAGAGC

SP40 TTATTAGTGGTGGTGGTGGTGGTGTGAAAGCTTAGAACGCAGGTAG

SP41 CATACAGCAGTTGATGATAAGCACCACCACCACCACCACTGAG

SP42 CATGAATGGTCTTCGGTTTCCG

SP43 CGGAAACCGAAGACCATTCATG

SP44 CTCAGTGGTGGTGGTGGTGGTGCTTATCATCAACTGCTGTATG

SP45	GGCCTGGTGCCGCGCGGCAGCAGTCGATATCAGCATACTAAA
SP46	GGCTTTGTTAGCAGCCGGATCTTAGTGATTTACTTTCTTGCCACT
SP47	GATCCGGCTGCTAACAAAGCC
SP48	GCTGCCGCGCGGCACCAGGCC
SP49	GTGCGGATCTCACCAACAGATTCATCAGGCCGCCGC
SP50	GCGGCGGCCTGATGAATCTGTTGGTGAGATCCGCACAAG
SP51	GGCCTGGTGCCGCGCGGCAGCGAATTATCAGAAATTAGAGCAG
SP52	AGGTCAAGAGACCCCCTAAAGTCCGC
SP53	AGGGGGTCTCTTGACCTCGAATCAAAGGA
SP54	GGCTTTGTTAGCAGCCGGATCTTATGAAAGCTTAGAACGCAG
SP55	TATAAAGACGACGACGACAAATAGAGATCCGATCAGACCAGT
SP56	GTCGTCGTCGTCTTTATAGTCGGCGGCTTTTTGGGGCACAAAAAAATC
SP57	GTCGTCGTCGTCTTTATAGTCCTTATCATCAACTGCTGTATG
SP58	ACCATACAGCAGTAGACGACAAAGACTTCTTCGTGCCCCAAAAAG
SP59	TCGTCTACTGCTGTATGGTCGTACGGGTTCTTATGCTTCCGTTTAT
SP60	GCTCGTGTTGTACAATAAATGTAGGAATCCTTAAGGTTTACGGTTTTAGAGCTAGAAATAGC AAGTTAAAATAAGGC
SP61	ACATTTATTGTACAACACGAGCC
SP62	TTCGATAAGCTTCTAGGATCCCATGCAGCTCTTACAGCAGTG
SP63	GGCCAAAAAACTGCTGCCTTCCTAGGCGGCTTTTTGGGGCAC
SP64	GAAGGCAGCAGTTTTTTGGCCTTC
SP65	GGATCCTAGAAGCTTATCGAA
SP66	GCTCGTGTTGTACAATAAATGTGTGTTTACGAGATCGCCTCTGTTTAGAGCTAGAAATAGC AAGTTAAAATAAGGC
SP67	GGCGCGGGCGCACAGCACGTCAATACGACG
SP68	GCCCGCGCCGCTTGCACGGTA
SP69	GATCCTAGAAGCTTATCGAATTCC
SP70	GCAGTCTAGACTCGAGTAAGG
SP71	CCTTACTCGAGTCTAGACTGCTCGAATTCTCATGTTTGACAG
SP72	GGAATTCGATAAGCTTCTAGGATCCGATCA
SP73	TTAACTAATAAGGAGGACAAACATGTCCAATTTACTGACCGT
SP74	CCGGTTATTATTACTAATCGCCATCTTCCA
SP75	CGATTAGTAATAATAACCGGGCAGGCCATG
SP76	TTGTCCTCCTTATTAGTTAATCAATTCAAGCTTAATTGTTAT
SP77	AAGCTTGGCGTAATCATGGTC
SP78	GAGCTCGAATTCCTGCAGCTG
SP79	GGTACCCGGGGATCCACTAGT
SP80	GCATGCCTCGAGGGGCCGCCC

SP81	CAGCTGCAGGAATTCGAGCTCATCTCACCCGCCACTGCTTTT
SP82	ACTAGTGGATCCCCGGGTACCCACATTTTCACCTTTCTTTGA
SP83	GGGCGGCCCCTCGAGGCATGCTTCTTTGTATCGAATCAGCTT
SP84	GACCATGATTACGCCAAGCTTTTTTCCGGTCCGTTTTGACAG
SP85	GTGAATTCGATAAGCTTCTAGAAGCTAGGAGGAGGATGTGATGACAATGTTTGT
SP86	CACACAAATTAAAAACTGGTCT
SP87	AGACCAGTTTTTAATTTGTGTG
SP88	TAGAAGCTTATCGAATTCAC
SP89	GGTACCCGGGGATCCACTAGT
SP90	GCATGCCTCGAGGGGCCGCCC
SP91	CAGCTGCAGGAATTCGAGCTCTAAATTGACAATGCAGTCCAG
SP92	ACTAGTGGATCCCCGGGTACCATTCTCCTCCTTGTTCTCTTA
SP93	GGGCGGCCCCTCGAGGCATGCGCTGATGCTCCGCTCGATATG
SP94	GACCATGATTACGCCAAGCTTATTTCCATGCCCATCGCCATC
SP95	TAACTTTAAGAAGGAGGGAGATATACCAATGACAATGTTTGTGGGATC
SP96	TTTGTATAGTTCATCCATGCC
SP97	TTATTATAAAAGAAGAGAACC
SP98	GAAATTAATACGACTCACTATAGGGAGACCACAACGGTTTCCCTCTAGAAATAATTTTGTTT AACTTTAAGAAGGAG
SP99	TAACTTTAAGAAGGAGGATTTTAGAATGACCATGATTACGGATTCA
SP100	AAAACTGGTCTGATCGGATCTTTATTTTGACACCAGACCAACTG
SP101	AGATCCGATCAGACCAGTTTT
SP102	AAATCCTCCTTCTTAAAGTTACTATTTGTCGTCGTCGTCTTT
nlpD rev	CGGCGGTCTAATCAACATAC

Supplementary Table 6.

Plasmid name	Combinations of primers and template DNAs for plasmid construction					
pNAR756	SP1, 2	/PY79 DNA	SP3, 4	/pCH1141		
pNAR758	SP5, 6	/pNAR756				
pNAR778	SP7, 8	/pNR1	SP9, 10	/pNAR756		
pNAR780	SP7, 11	/pNR1	SP10, 12	/pNAR758		
pNAR809	SP13, 14	/PY79 DNA	SP15, 16	/pLOSS*		
pNAR813	SP17, 18	/pNAR809				
pNAR869	SP19, 20	/PY79 DNA	SP21, 22	/pET28b		
pNAR879	SP23, 24	/PY79 DNA	SP25, 26	/pCH913		
pNAR901	SP27, 28	/PY79 DNA	SP29, 30	/pCH1142		

pNAR903	SP31, 32	/PY79 DNA	SP33, 34	/pNAR901				
pNAR913	SP35, 36	/PY79 DNA	SP37, 38	/pET28b				
pNAR915	SP39, 40	/pCH2238	SP37, 38	/pET28b				
pNAR916	SP41, 42	/pNAR869	SP43, 44	/pNAR869				
pNAR917	SP45, 46	/JM109 DNA	SP42, 47	/pET28b	SP43, 48	/pET28b		
pNAR939	SP49, 50	/pNAR915						
pNAR1036	SP99, 100	/pCH1517	SP101, 102	/pCH2239				
pNAR1037	SP99, 100	/pCH1517	SP101, 102	/pCH2240				
pNAR1038	SP99, 100	/pCH1517	SP101, 102	pCH2241				
pCH2238	SP51, 52	/PY79 DNA	SP53, 54	/PY79 DNA	SP47, 48	/pET28b		
pCH2239	SP55, 56	/pNAR879						
pCH2240	SP55, 57	/pNAR879						
pCH2241	SP58, 59	/pCH2239						
pCH2264	SP60, 61	/pKIG855						
pCH2293	SP62, 63	/PY79 DNA	SP64, 65	/pDG1731				
pCH2302	SP61, 66	/pKIG855						
pCH2307	SP67, 68	/pNAR915						
pKIG855	SP69, 70	/synthetic DNA	SP71, 72	/pDG1662				
pMK2	SP73, 74	/P1 phage DNA	SP75, 76	/pLOSS*				
pKY13	SP77, 78	/pCH1142	SP79, 80	/pCH1142	SP81, 82	/PY79 DNA	SP86, 87	/PY79 DNA
pNR1	SP85, 86	/pCH747	SP87, 88	/pCH1130				

Supplementary Table 7. PCR templates and primers for preparation of in vitro translation templates

Gene name	1st PCR			2nd PCR		
	Primers		template	Primers		template
GFP-ns	SP95	SP96	pCH805	SP98	SP96	1st PCR product
GFP-*mifM*	SP95	SP97	pCH805	SP98	SP97	1st PCR product

Supplementary Figures

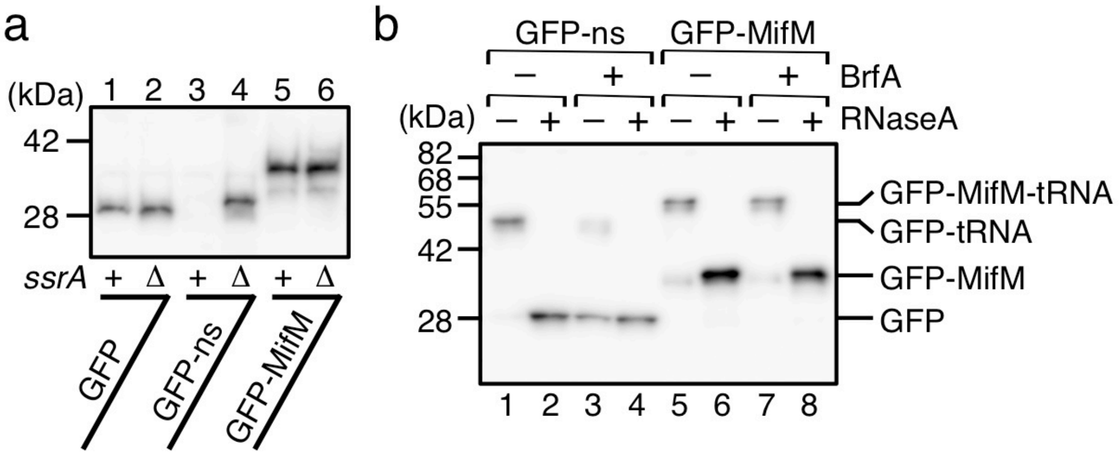

Supplementary Fig. 1 | Nascent-chain mediated ribosome stalling is refractory to the ribosome rescue systems in *B. subtilis*. **a**, MifM is refractory to *trans*-translation. Cellular accumulation of GFP-MifM was examined in the wild type and *ssrA*-deficient mutant strains. GFP (lanes 1, 2), GFP-ns (lanes 3, 4) and GFP-MifM (lanes 5, 6) were expressed in the wild type (odd number) and Δ*ssrA* (even number) strains of *B. subtilis*, separated by SDS-PAGE and detected with anti-GFP immunoblotting. **b**, Inability of BrfA to induce RF2 hydrolysis of the elongation-arrested MifM-tRNA. In vitro translation using *Bs* hybrid PURE system with RF2 was directed by the *gfp-ns* (lanes 1-4) or *gfp-mifM* (lanes 5-8) template in the presence (lanes 3, 4, 7, 8) or the absence (lanes 1, 2, 5, 6) of purified BrfA62-His$_6$. The translation products were analyzed by anti-GFP immunoblotting as described above.

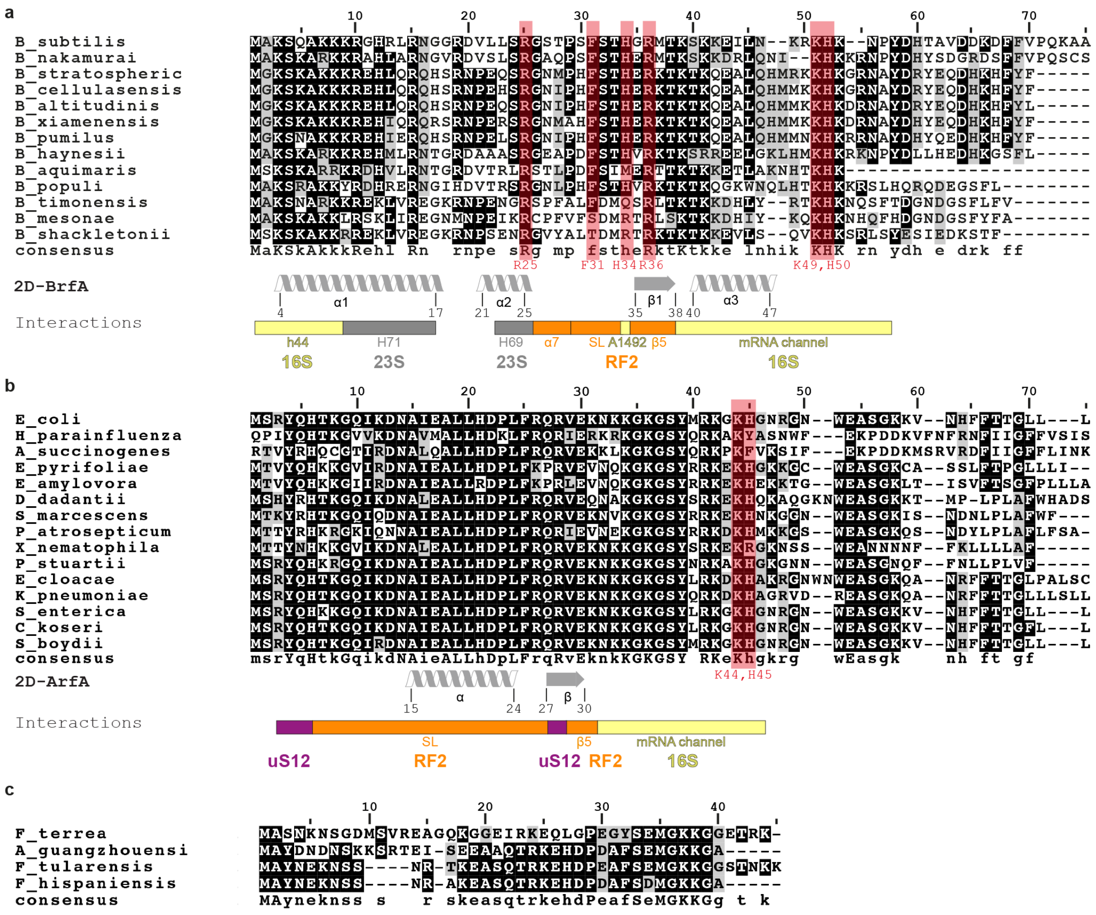

Supplementary Fig. 2 | Multiple sequence alignments of BrfA, ArfA, and ArfT homologs. **a-c**, Multiple sequence alignments of (**a**) BrfA, (**b**) ArfA and (**c**) ArfT generated with Clustal W[11] and Boxshade with residues shaded that are identical (black) or similar (grey). Below each alignment, the consensus sequence with capital letters indicating 100% conservation and lower-case letters indicating less conservation). The known secondary structure is shown for *B. subtilis* BrfA (2D-BrfA) in (**a**) and *E. coli* ArfA (2D-ArfA) in (**b**), as well as interaction regions between (**a**) BrfA and (**b**) ArfA with the ribosome (16S rRNA, yellow, 23S rRNA, grey and uS12, violet) and RF2 (orange).

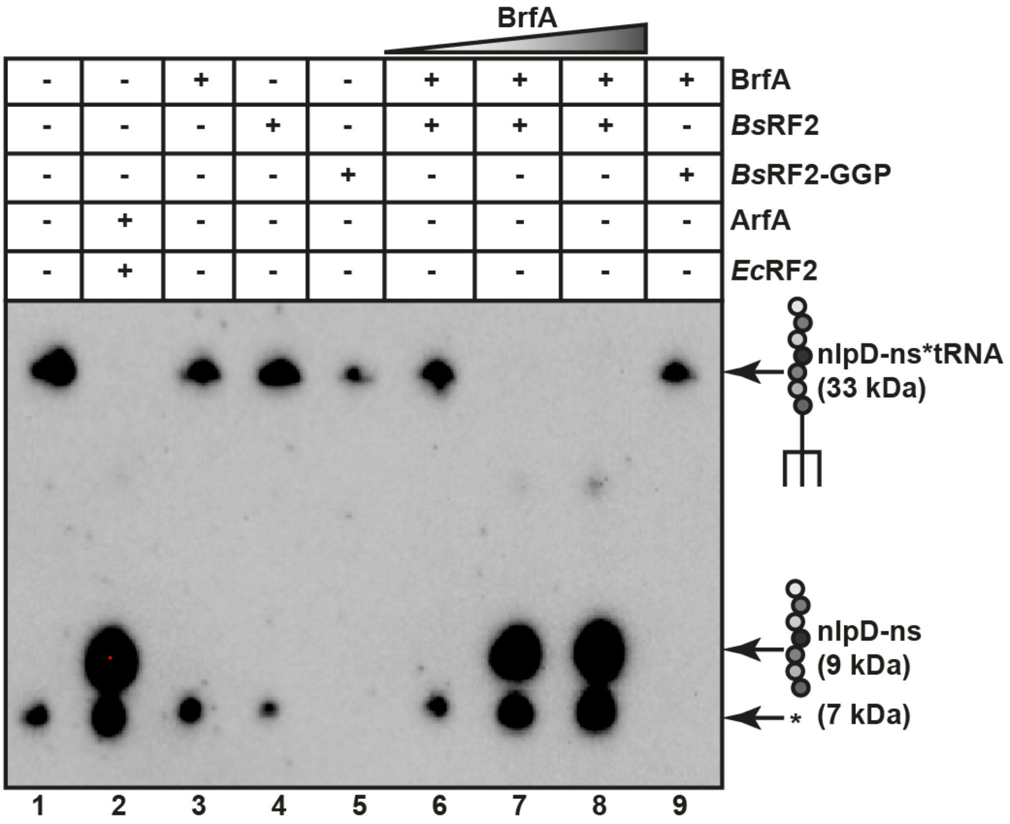

Supplementary Fig. 3 | Recycling of non-stop ribosomes by BrfA and RF2.

In vitro translation assays using the *E. coli* PURE systemΔRF123 kit (lacking all RFs) were performed with truncated non-stop *nlpD* DNA template, in the presence of BrfA, wildtype *B. subtilis* RF2 (*Bs*RF2) and the RF2-GGP mutant (*Bs*RF2-GGP) alone (lanes 3-5), or *Bs*RF2 (20 pmol) with increasing concentrations (12-100 pmol) of BrfA (lanes 6-8), or BrfA (100 pmol) in the presence of 100 pmol *Bs*RF2-GGP (lane 9). As a positive control, reactions were performed with *E. coli* ArfA and RF2 (*Ec*RF2), as described previously (lane 2) [12]. A negative control was also performed where reactions lacked all RFs and rescue factors (lane 1). Western blotting of NuPAGE gels using an antibody against the HA-tag present in the N-terminus of the NlpD peptide detected the presence of the non-stop NlpD-peptidyl-tRNA (33 kDa) and released NlpD peptide (9 kDa). The asterisk (*) indicates a mysterious band that cross-reacts with the HA-antibody, but is also present in the negative control and was therefore not examined further. As expected, the negative control (lane 1), as well as reactions performed in the presence of BrfA, *Bs*RF2 or *Bs*RF2GGP alone (lanes 3-5), show a strong band for the NlpD-peptidyl-tRNA and no evidence for the released NlpD peptide. By contrast, the positive control with ArfA and *Ec*RF2 (lane 2), as well as the reactions with *Bs*RF2 and increasing concentrations of BrfA (lanes 7 and 8) shows no NlpD-peptidyl-tRNA and only the presence of released NlpD peptide. As expected, substitution of wildtype *Bs*RF2 with the inactive *Bs*RF2-GGP mutant led to a loss the band for the released NlpD peptide and the presence of NlpD-peptidyl-tRNA was restored (lane 9). Source data are provided as a Source Data file.

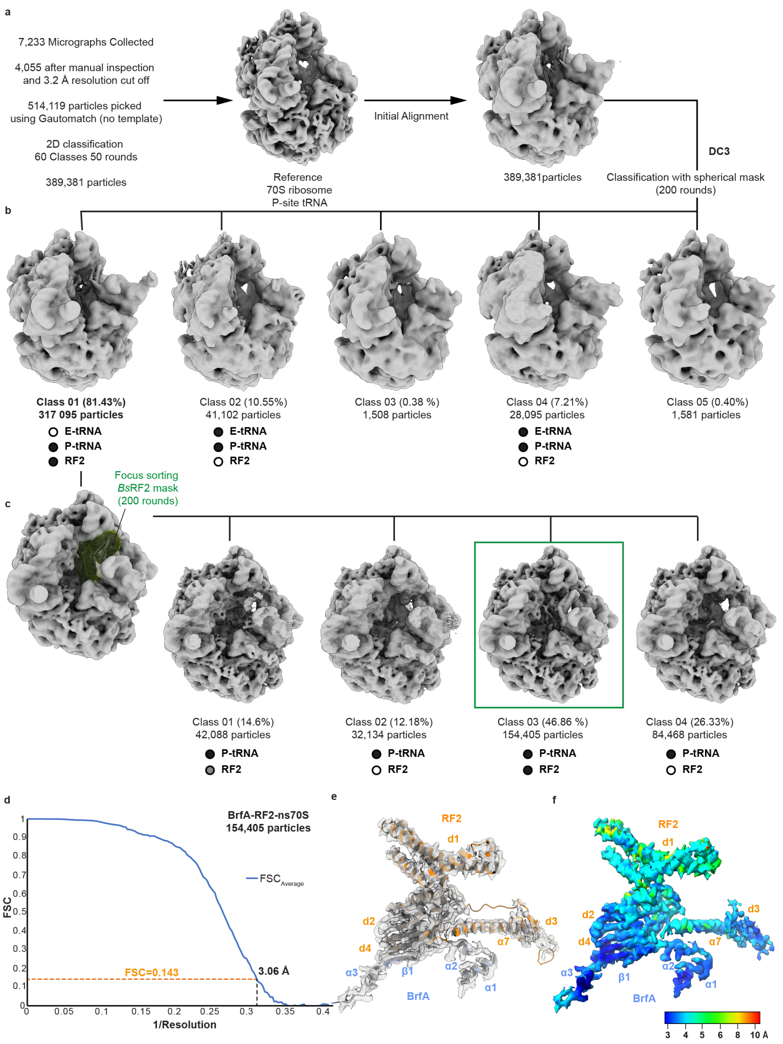

Supplementary Fig. 4 | *In silico* sorting of the BrfA-RF2-ns70S complex.

a, After particle picking and extensive 2D classification, the complete dataset of 389,381 particles was initially aligned against a P-site tRNA containing *E. coli* 70S ribosome. **b**, Following 3D classification for 200 rounds in Relion, five classes were generated. The majority (317,095 particles; 81.43%) of particles were found in class 1 and contained the BrfA-RF2-ns70S complex. The second major (41,102 particles; 10.55%) class 2 contained a fully programmed ribosome, but without the presence of RF2. In addition, three minor classes 4-6 (class 3; 1,508 particles; 0.38 %, class 4; 28,095 particles; 7.21%; class 5; 1,581 particles; 0.4%) containing damaged and/or poorly aligning particles were observed. **c**, The 317,095 particles from the class 1 were further sorted using a focus sorting mask around RF2, resulting in four additional classes, of which class 3 (154,405 particles, 46.86%) contained stoichiometric occupancy of P-site tRNA, RF2 and BrfA. Class 3 was then refined to yield a (**d**) final reconstruction of the BrfA-RF2-ns70S complex with an average resolution of 3.15 Å (0.143 FSC). **e-f**, Isolated electron density for BrfA and RF2 **e**, shown as grey mesh with fitted model and (**f**) colored according to local resolution

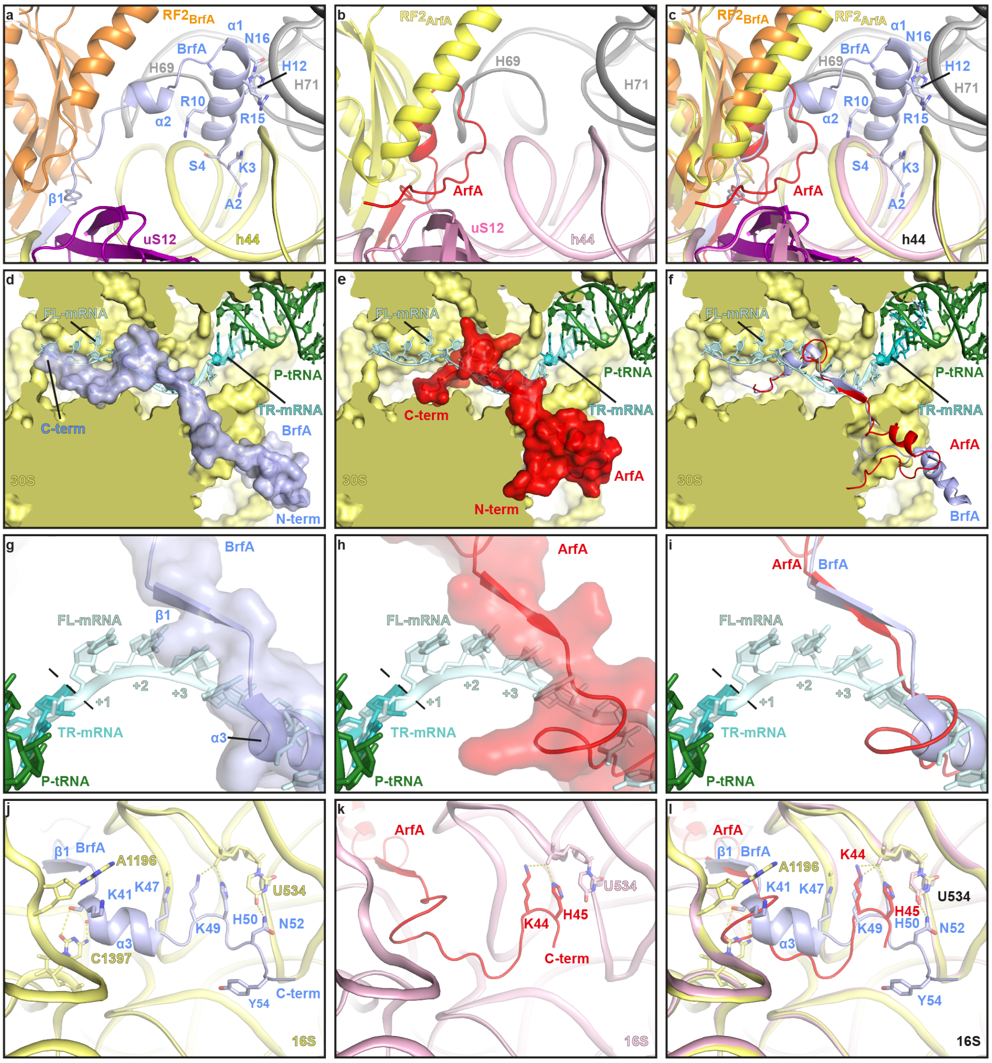

Supplementary Fig. 5 | Interactions of BrfA and ArfA on the ribosome. **a**, Interaction of the N-terminus of BrfA (blue) with RF2 (orange), uS12 (violet), helix 44 (h44) of the 16S rRNA (yellow) and helix 69 (H69) and H71 of the 23S rRNA (grey). **b**, Same view as (**a**), but for the ArfA-RF2-70S complex, with ArfA (red), RF2 (yellow) and 16S rRNA (pink) [12]. **c**, Overlay of (**a**) and (**b**). **d-e**, Transverse section of the 30S subunit (yellow) to reveal the mRNA channel showing a superimposition of full-length mRNA (FL-mRNA, cyan) with truncated non-stop mRNA (TR-mRNA, teal), P-site tRNA (green) and surface representations of (**d**) BrfA (blue) and (**e**) ArfA (red) [12]. **f**, Overlay of (**d**) and (**e**) with cartoon representations of BrfA (blue) and ArfA (red). **g-h**, Superimposition of full-length mRNA (FL-mRNA, cyan) with truncated non-stop mRNA (TR-mRNA, teal), P-site tRNA (green) and transparent surface representations of (**g**) BrfA (blue) and (**h**) ArfA (red) [12]. The first (+1), second (+2) and third (+3) nucleotides of the A-site codon of the FL-mRNA are indicated. **i**, Overlay of (**g**) and (**h**) with cartoon representations of BrfA (blue) and ArfA (red). **j-k**, Interaction of the C-terminus of (**j**) BrfA (blue) and (**k**) ArfA (red) [12] with the 16S rRNA with potential hydrogen bonds indicated with yellow dashed lines. **l**, Overlay of (**j**) and (**k**).

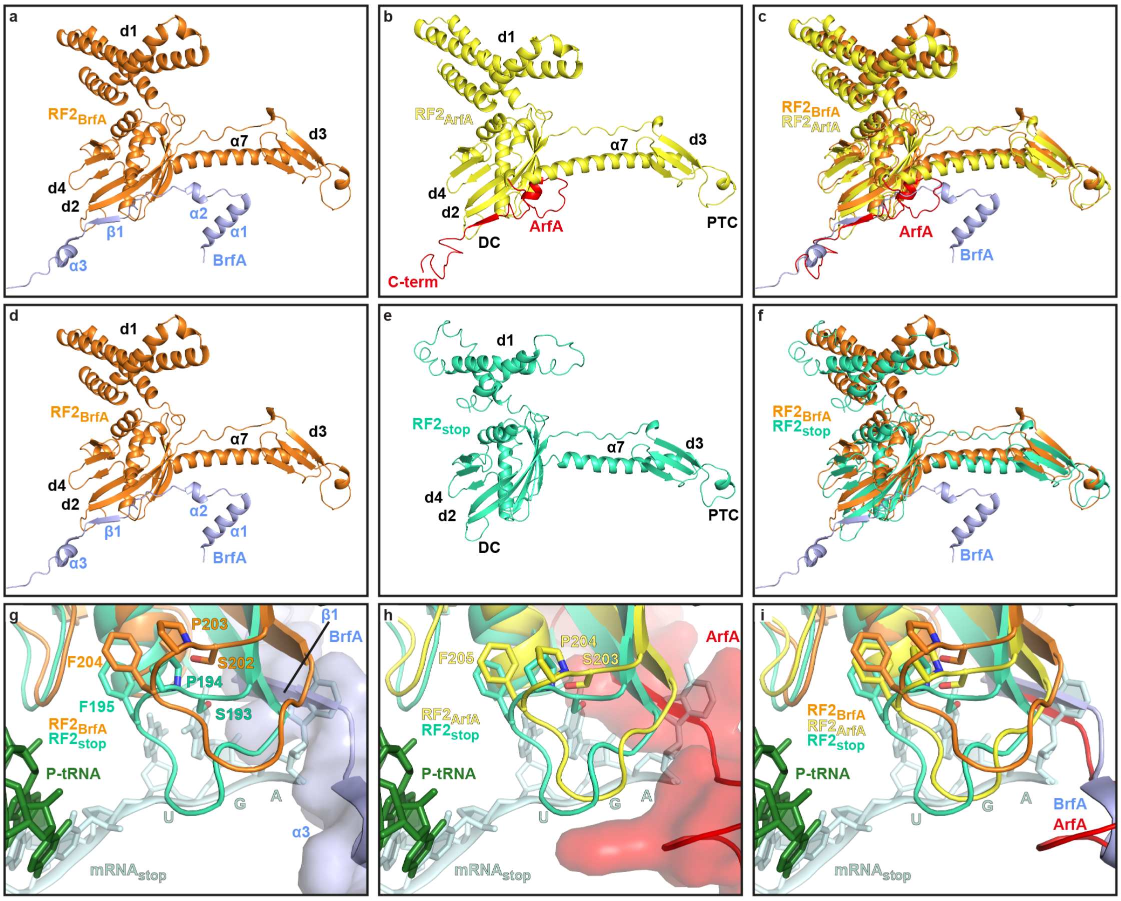

Supplementary Fig. 6 | Interaction of BrfA and ArfA with RF2 on the ribosome.

a-b, Interaction between (**a**) BrfA (blue) and $RF2_{BrfA}$ (orange) in the BrfA-RF2-ns70S complex compared with (**b**) ArfA (red) and $RF2_{ArfA}$ (yellow) in the ArfA-RF2-ribosome complex [12]. The approximate positions of the decoding center (DC) and peptidyltransferase center (PTC) on the ribosome are indicated. **c**, Superimposition of (**a**) and (**b**). **d-e**, The binding position of (**d**) $RF2_{BrfA}$ (orange) and BrfA (blue) in the BrfA-RF2-ns70S complex, compared with (**e**) $RF2_{stop}$ (lime) in a canonical termination complex [13]. The approximate positions of the decoding center (DC) and peptidyltransferase center (PTC) on the ribosome are indicated. **f**, Superimposition of (**d**) and (**e**). **g-h**, Superimposition of the SPF motif of $RF2_{stop}$ (lime) and UGA codon of the mRNA (cyan) (PDB ID 4V5E, [13]) with (**g**) the SPF motif of $RF2_{BrfA}$ (orange) and BrfA (blue), and (**h**) with the SPF motif of $RF2_{ArfA}$ (yellow) and ArfA (red) [12]. **i**, Overlay of (**g**) and (**h**) with BrfA (blue) and ArfA (red) as cartoon representations.

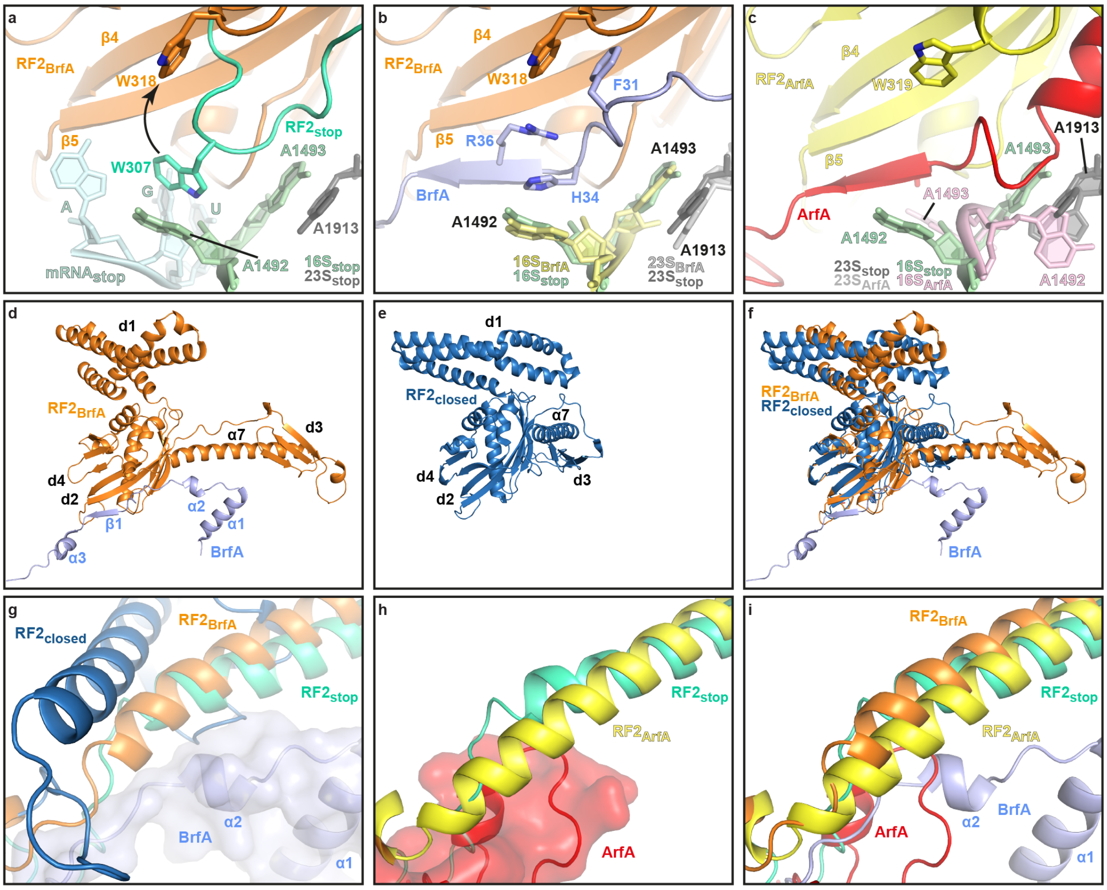

Supplementary Fig. 7 | BrfA induces an open conformation of RF2 on the ribosome.

a, Interaction between Trp307 (W307, which is equivalent to *B. subtilis* Trp318 (W318)) of the switch region of *Thermus thermophilus* RF2$_{stop}$ (lime) and A1492 of the 16S rRNA (green) during decoding of the UGA stop codon of the mRNA (cyan; PDB ID 4V5E[13]). W318 in the switch loop of RF2 (RF2$_{BrfA}$, orange) observed upon BrfA binding is superimposed and arrowed. **b**, Same view as in (**a**), showing the conformation of the switch loop of RF2$_{BrfA}$ (orange) and A1492/A1493 (pale yellow) when BrfA (blue) is present. **c**, Same view as in (**a**) and (**b**), showing the conformation of A1492/A1493 in comparison to (**a**) and the switch loop in the presence of ArfA (red, PDB ID 5MVGP[12]). (**d**) Open conformation observed for RF2$_{BrfA}$ (orange) when in complex with BrfA on the ribosome, compared with (**e**) the closed conformation of RF2$_{closed}$ (dark blue, PDB ID 1GQE[14]) when not bound to the ribosome. **f**, Superimposition of (**d**) and (**e**). **g**, Superimposition of the conformation of helix α7 of RF2 from the crystal structure of the closed form of RF2$_{closed}$ with RF2$_{Stop}$ (lime) and RF2$_{BrfA}$ (orange), with BrfA (blue) shown for reference. **h-i**, Same view as in (**g**) showing the conformation of helix α7 of RF2$_{stop}$ superimposed with (**h**) RF2$_{ArfA}$, and ArfA (red) and (**i**) including RF2$_{BrfA}$ (orange) and BrfA (blue).

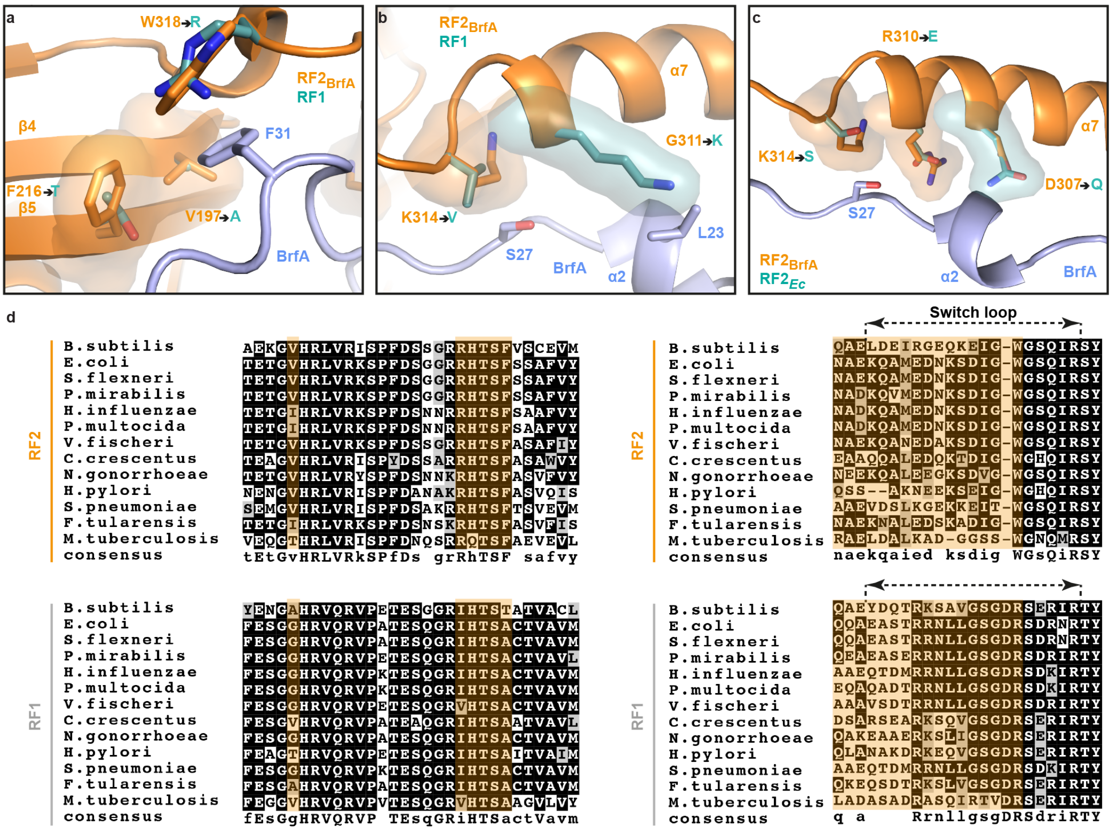

Supplementary Fig. 8 | Basis for the species-specificity of BrfA

a-b, Potential species-specific interactions between BrfA (blue) and *B. subtilis* RF2 (orange) compared with a homology model for *B. subtilis* RF1 (green) aligned to the BrfA-RF2-ns70S. **a**, The BrfA interface with β4 and β5 strands of *B. subtilis* RF2 (orange) consists of hydrophobic residues Val197 and Phe216, which are substituted by Ala and Thr, respectively in *B. subtilis* RF1. **b**, The BrfA interface with the switch loop and helix α7 of *B. subtilis* RF2 (orange) consists of multiple residues that are distinct *B. subtilis* RF2 and RF1, for example, G311, K314 and W318 (seen in (**a**)) of RF2 that are substituted with Lys, Val and Arg, respectively, in *B. subtilis* RF1. **c**, Potential sequence differences between *B. subtilis* RF2 (orange) and *E. coli* RF2 (green) that could come within close proximity of BrfA and could explain the species-specific activity of BrfA. **d**, Sequence alignments of RF1 and RF2 for the corresponding regions shown in (**a**)-(**c**).

Supplementary References

1 Youngman, P., Perkins, J. B. & Losick, R. A novel method for the rapid cloning in Escherichia coli of Bacillus subtilis chromosomal DNA adjacent to Tn917 insertions. *Mol Gen Genet* **195**, 424-433, (1984).

2 Koo, B. M. *et al.* Construction and Analysis of Two Genome-Scale Deletion Libraries for Bacillus subtilis. *Cell Syst* **4**, 291-305 e297, (2017).

3 Muto, A. *et al.* Requirement of transfer-messenger RNA for the growth of Bacillus subtilis under stresses. *Genes Cells* **5**, 627-635, (2000).

4 Chiba, S., Lamsa, A. & Pogliano, K. A ribosome-nascent chain sensor of membrane protein biogenesis in Bacillus subtilis. *EMBO J* **28**, 3461-3475, (2009).

5 Steinmetz, M. & Richter, R. Plasmids designed to alter the antibiotic resistance expressed by insertion mutations in Bacillus subtilis, through in vivo recombination. *Gene* **142**, 79-83, (1994).

6 Guerout-Fleury, A. M., Frandsen, N. & Stragier, P. Plasmids for ectopic integration in Bacillus subtilis. *Gene* **180**, 57-61, (1996).

7 Claessen, D. *et al.* Control of the cell elongation-division cycle by shuttling of PBP1 protein in Bacillus subtilis. *Mol Microbiol* **68**, 1029-1046, (2008).

8 Peters, J. M. *et al.* A Comprehensive, CRISPR-based Functional Analysis of Essential Genes in Bacteria. *Cell* **165**, 1493-1506, (2016).

9 Saito, A. *et al.* Post-liberation cleavage of signal peptides is catalyzed by the site-2 protease (S2P) in bacteria. *Proc Natl Acad Sci U S A* **108**, 13740-13745, (2011).

10 Sohmen, D. *et al.* Structure of the Bacillus subtilis 70S ribosome reveals the basis for species-specific stalling. *Nat Commun* **6**, 6941, (2015).

11 Larkin, M. A. *et al.* Clustal W and Clustal X version 2.0. *Bioinformatics* **23**, 2947-2948, (2007).

12 Huter, P. *et al.* Structural basis for ArfA-RF2-mediated translation termination on mRNAs lacking stop codons. *Nature* **541**, 546-549, (2017).

13 Weixlbaumer, A. *et al.* Insights into translational termination from the structure of RF2 bound to the ribosome. *Science* **322**, 953-956, (2008).

14 Vestergaard, B. *et al.* Bacterial polypeptide release factor RF2 is structurally distinct from eukaryotic eRF1. *Mol Cell* **8**, 1375-1382, (2001).
