# Supplementary material for: Release factor-dependent ribosome rescue by BrfA in the Gram-positive bacterium Bacillus subtilis
Source: Nat Commun. 2019 Nov 27;10:5397. doi: 10.1038/s41467-019-13408-7 (PMC6881298; doi:10.1038/s41467-019-13408-7)
Supplement: Supplementary file 3 — Reporting Summary [file 41467_2019_13408_MOESM3_ESM.pdf]

nature research

Corresponding author(s): Shinobu Chiba and Daniel Wilson

Last updated by author(s): Oct 3, 2019

Reporting Summary

Nature Research wishes to improve the reproducibility of the work that we publish. This form provides structure for consistency and transparency in reporting. For further information on Nature Research policies, see Authors & Referees and the Editorial Policy Checklist .

Statistics

For all statistical analyses, confirm that the following items are present in the figure legend, table legend, main text, or Methods section.

n/a	Confirmed	
☐	☒	The exact sample size (*n*) for each experimental group/condition, given as a discrete number and unit of measurement
☐	☒	A statement on whether measurements were taken from distinct samples or whether the same sample was measured repeatedly
☐	☒	The statistical test(s) used AND whether they are one- or two-sided<br>*Only common tests should be described solely by name; describe more complex techniques in the Methods section.*
☒	☐	A description of all covariates tested
☒	☐	A description of any assumptions or corrections, such as tests of normality and adjustment for multiple comparisons
☒	☐	A full description of the statistical parameters including central tendency (e.g. means) or other basic estimates (e.g. regression coefficient) AND variation (e.g. standard deviation) or associated estimates of uncertainty (e.g. confidence intervals)
☒	☐	For null hypothesis testing, the test statistic (e.g. *F*, *t*, *r*) with confidence intervals, effect sizes, degrees of freedom and *P* value noted<br>*Give P values as exact values whenever suitable.*
☒	☐	For Bayesian analysis, information on the choice of priors and Markov chain Monte Carlo settings
☒	☐	For hierarchical and complex designs, identification of the appropriate level for tests and full reporting of outcomes
☒	☐	Estimates of effect sizes (e.g. Cohen's *d*, Pearson's *r*), indicating how they were calculated

Our web collection on statistics for biologists contains articles on many of the points above.

Software and code

Policy information about availability of computer code

Data collection	Amersham Imager 600 ver. 1.1.1 for Western blotting. Cryo-EM data were collected using Titan Krios-TEM (FEI, Netherland), EM-TOOLS (TVIPS GmbH) and EPU (FEI, Netherland).
Data analysis	Cryo-EM: Gautomatch, MotionCor2, CTFFIND3, GCTF v1.06, Relion 3, SPHIRE, Molprobity, Coot, Phenix, UCSF Chimera, PyMol Molecular Graphic Systems (Version 1.8 Schrödinger).

For manuscripts utilizing custom algorithms or software that are central to the research but not yet described in published literature, software must be made available to editors/reviewers. We strongly encourage code deposition in a community repository (e.g. GitHub). See the Nature Research guidelines for submitting code & software for further information.

Data

Policy information about availability of data

All manuscripts must include a data availability statement. This statement should provide the following information, where applicable:

- Accession codes, unique identifiers, or web links for publicly available datasets
- A list of figures that have associated raw data
- A description of any restrictions on data availability

All data that support the findings of this study are available from the corresponding authors upon reasonable request. Cryo-EM maps and associated models will be released in the EMDB (EMD-10353) and PDB (6SZS) upon acceptance.

Field-specific reporting

Please select the one below that is the best fit for your research. If you are not sure, read the appropriate sections before making your selection.

- [x] Life sciences
- [ ] Behavioural & social sciences
- [ ] Ecological, evolutionary & environmental sciences

For a reference copy of the document with all sections, see nature.com/documents/nr-reporting-summary-flat.pdf

Life sciences study design

All studies must disclose on these points even when the disclosure is negative.

Sample size	We repeated Western blotting, b-galactosidase assay and bacterial growth assay at least twice using samples or cultures independently prepared in different days.
Data exclusions	For cryo-EM, 2D and 3D classification was performed in RELION to sort particles into individual subpopulations. Subpopulations lacking the ligands of interest were not analyzed further
Replication	All attempts to reproduce data for in vivo and in vitro assays were successful.
Randomization	We randomly selected bacterial colonies for in vivo analysis. For cryo-EM, unsupervised classification was used to assign particles to particular subclasses during 3D classification in RELION
Blinding	Samples were numbered, analyzed and then linked back to the type of protein or bacterial strain for drawing the conclusions. For cryo-EM, the 3D classification is unsupervised therefore the researcher does not influence the allocation of particles to the particular subclasses.

Reporting for specific materials, systems and methods

We require information from authors about some types of materials, experimental systems and methods used in many studies. Here, indicate whether each material, system or method listed is relevant to your study. If you are not sure if a list item applies to your research, read the appropriate section before selecting a response.

Materials & experimental systems

n/a	Involved in the study	
[ ]	[x]	Antibodies
[x]	[ ]	Eukaryotic cell lines
[x]	[ ]	Palaeontology
[x]	[ ]	Animals and other organisms
[x]	[ ]	Human research participants
[x]	[ ]	Clinical data

Methods

n/a	Involved in the study	
[x]	[ ]	ChIP-seq
[x]	[ ]	Flow cytometry
[x]	[ ]	MRI-based neuroimaging

Antibodies

Antibodies used	anti-GFP, anti-FLAG, anti-HA
Validation	Specificity of antibodies were confirmed by Western blotting using negative control samples.
