# Supplementary material for: Release factor-dependent ribosome rescue by BrfA in the Gram-positive bacterium Bacillus subtilis
Source: Nat Commun. 2019 Nov 27;10:5397. doi: 10.1038/s41467-019-13408-7 (PMC6881298; doi:10.1038/s41467-019-13408-7)
Supplement: Supplementary file 2 — Peer Review [file 41467_2019_13408_MOESM2_ESM.pdf]

Reviewers' comments:

Reviewer #1 (Remarks to the Author):

All cells have quality control mechanisms that rescue and recycle ribosomes stalled on mRNA transcripts. In bacteria, the trans-translation mechanism is widely conserved (involving tmRNA) but there are also backup systems in many bacteria that are poorly conserved. In this manuscript, Chiba and co-workers identify an alternate rescue factor ResQ in B. subtilis using a synthetic lethal screen. They show that ResQ expression depends on tmRNA activity, consistent with its role as a backup ribosome rescue factor. In vitro biochemical studies show that ResQ recruits RF2 (and not RF1) to stalled ribosomes to promote peptidyl hydrolysis. Finally, in collaboration with Wilson and co-workers, they solve the cryo-EM structure of ResQ and RF2 in the A site showing how it binds selectively to stalled ribosomes (through its interaction with the mRNA channel) and how it promotes a conformation in RF2 that activates hydrolysis. I find the data supporting these claims to be compelling. This manuscript reports the whole story from beginning to end (it is a real tour de force); the writing is clear and well argued. Appropriate comparisons and contrasts are made to ArfA, a well-studied ribosome rescue factor in E. coli that also works by recruiting RF2 to stalled ribosomes.

I have two suggestions to further strengthen the paper:

1) Several places in the manuscript the authors make the strong claim that ResQ is synthesized from a non-stop transcript. This is shown indirectly but never formally proven, merely inferred from the prediction of a rho-independent terminator. The authors should determine the 3'-end of the mRNA using RACE in an ssrA or smpB knockout strain. I would like to know exactly where the mRNA is truncated and how long the ResQ protein is in its natural context.

2) While the authors' new name for ResQ is cute, to be sure, I confess that I am sorry to see them depart from the consistent alternate rescue factor (arf) nomenclature. ArfA and ArfT also work by recruiting RFs to stalled ribosomes; why not call this protein ArfS where S stands for subtilis?

Reviewer #2 (Remarks to the Author):

The manuscript by Chiba and Wilson reported the discovery and mechanistic dissection of an alternative ribosome rescue factor in Gram-positive bacterial species. Overall, this is a very well executed study, with convincing data from bacterial genetics, biochemistry and structural biology. I recommend the publication of this manuscript after addressing the following issues.

While the majority of data analysis and interpretation is solid, a major concern is that the authors tended to claim ResQ being an independently evolved rescue factor. Since ResQ and ArfA are both small proteins, and their interactions with the ribosome (and RF2) are mostly not very much sequence-specific, it is highly likely they are actually products of divergent evolution. From a structural point of view, although detailed interactions between the factor and the ribosome (and between RF2 and the factor) are different in some regions for ArfA and ResQ, they display high similarities in many aspects: for example, the beta-strand interaction with RF2; two structurally conserved C-terminal residues (His and Lys); the ability to interact and change the switch region of RF2; and the binding of N-terminus in H69-H44 region. Therefore, it is hard to imagine that they do not have a common ancestor.

A few minor issues:

1. Line 239 GAQ mutation of RF2 no longer stimulated the hydrolysis of GFP-tRNA in the presence of ResQ. Is there special consideration that GGP mutation, instead of GAQ was used to perform cryo-EM

analysis (line299)?.

2. U1915 mentioned in line 324 was not labeled in fig. 4f.

3. There appear to be duplicate panels between Figure 4 and Supplementary Fig. 5, between Figure 5 and Supplementary Fig. 6, and between Figure 5 and supplementary Fig. 7.

Reviewer #3 (Remarks to the Author):

In the manuscript by Shimokawa-Chiba and colleagues, the authors discover a new ribosome quality control pathway that serves as an essential backup to the trans-translation based ribosome quality control system. This new pathway, which uses ResQ (renamed from YqkK), is the first release factor (RF) dependent system described in a Gram-positive bacterium. First, the authors use a genome-wide selection strategy to identify genes that are essential in B. subtilis when the trans-translation system is inactive. They then verify the essentiality of ResQ in this context, using alternative knockdown and rescue strategies. The authors then carried out a range of molecular biology and biochemical experiments to establish the regulatory mechanism of ResQ expression, and how ResQ itself functions in an RF2-dependent manner. Additionally, the authors define the species-selectivity of the ResQ system, finding it depends on the species-specific RF2, but not the bacterial source of the ribosome. Finally, the authors use cryo-EM to determine the molecular basis for how ResQ recruits RF2 to stalled ribosome nascent-chain complexes, to define when RecQ is likely to function, i.e. on truncated mRNAs. It's particularly striking how the ResQ system has evolved by convergent evolution to harbor many of the features of the E. coli ArfA ribosome quality control system, even though the molecular details differ in key respects. This includes similarities to how ArfA expression is regulated. Overall, this is a really interesting paper, and will be of wide interest to the microbiology community as well as the translation field.

The paper is overall well written. The following points should be addressed by the reviewers as minor revisions.

1. Given the convergent evolution seen here, and the two kinds of regulatory mechanism for generating trans-translation sensitive expression of ResQ and ArfA, the authors could shorten some of the other discussion points a little, and add a paragraph on how the termination mechanisms used to regulate ResQ and ArfA expression could be used to potentially identify other ribosome quality control pathways in bacteria that serve as backup to the trans-translation system. This could be a fruitful way to identify sequence-divergent proteins that serve the same role as ResQ/ArfA and that are regulated in a similar manner. I think this would broaden the interest of the paper.

2. In many of the figure panels, the labels are light and hard to see. For example, in Figure 4 with ~blue-green labels on the yellow background of the 30S subunit, and some of the other light labels. There are a few cases in Figures 4, 5, Supplementary Figure 2a-b, and Supplementary Figures 5-7 where it may be necessary to use black lines to connect darker labels to the structural feature, rather than using light labels.

3. Line 219-220. Please provide a citation (58?) and/or methods for the neutral-pH SDS gels. These may not be familiar to most readers. Also provide this citation on line 657, if this is the protocol used. Otherwise, please add details of the protocol.

Point to point responses to the reviewers' comments
(Reviewers' comments are in *italic*).

Reviewer #1 (Remarks to the Author):

All cells have quality control mechanisms that rescue and recycle ribosomes stalled on mRNA transcripts. In bacteria, the trans-translation mechanism is widely conserved (involving tmRNA) but there are also backup systems in many bacteria that are poorly conserved. In this manuscript, Chiba and co-workers identify an alternate rescue factor ResQ in B. subtilis using a synthetic lethal screen. They show that ResQ expression depends on tmRNA activity, consistent with its role as a backup ribosome rescue factor. In vitro biochemical studies show that ResQ recruits RF2 (and not RF1) to stalled ribosomes to promote peptidyl hydrolysis. Finally, in collaboration with Wilson and co-workers, they solve the cryo-EM structure of ResQ and RF2 in the A site showing how it binds selectively to stalled ribosomes (through its interaction with the mRNA channel) and how it promotes a conformation in RF2 that activates hydrolysis. I find the data supporting these claims to be compelling. This manuscript reports the whole story from beginning to end (it is a real tour de force); the writing is clear and well argued. Appropriate comparisons and contrasts are made to ArfA, a well-studied ribosome rescue factor in E. coli that also works by recruiting RF2 to stalled ribosomes.

Response: We appreciate the positive evaluation of our work.

I have two suggestions to further strengthen the paper:
1) Several places in the manuscript the authors make the strong claim that ResQ is synthesized from a non-stop transcript. This is shown indirectly but never formally proven, merely inferred from the prediction of a rho-independent terminator. The authors should determine the 3′- end of the mRNA using RACE in an ssrA or smpB knockout strain. I would like to know exactly where the mRNA is truncated and how long the ResQ protein is in its natural context.

Response: We agree with the reviewer about the importance of providing direct evidence for the internal transcription termination in *brfA*. We considered 3′RACE experiments suggested by the reviewer, but this experimental approach

may not be free from artifacts that are introduced during sample manipulations. On the other hand, transcription termination could be stochastic to give heterogeneous mRNA ends. We realize that the essential bottom-line requirement here is to show that transcription of *brfA* is indeed terminated before the *brfA* stop codon *in vivo*. We therefore designed and carried out *in vivo* experiments that can address the issue. We placed a reporter *lacZ* gene downstream of the *brfA*-coding region in the format of transcriptional fusion, and compared β-galactosidase activities when *brfA* sequence is wild-type and defective in the putative terminator. The results of this new experiment are included in Fig. 2d, which show that the *lacZ* expression remains nominal with the intact terminator sequence but increased dramatically by the terminator mutations. Thus, the terminator sequence blocks transcription beyond *brfA*. It should be reminded that we have shown in the original manuscript that the translation product of the C-terminally tagged *brfA* derivative lacked the tag sequence unless the terminator sequence was disrupted. Taken together, we now have strong evidence that transcriptional termination within the *brfA*-cording region renders *trans*-translation-dependent repression of the *brfA* expression. As stated above, our conclusion is not affected by the precise information about the 3′-end(s) of the *brfA* transcript, determination of which we would like to leave for future studies.

2) While the authors' new name for ResQ is cute, to be sure, I confess that I am sorry to see them depart from the consistent alternate rescue factor (arf) nomenclature. ArfA and ArfT also work by recruiting RFs to stalled ribosomes; why not call this protein ArfS where S stands for subtilis?

Response: We realize, thanks to a bioRxiv reader, that *Bacillus subtilis* already contains the *res* genes, making it inappropriate to use *resQ* for *yqkK*. The reviewer's comment also forced us to rename this gene/protein. The suggested name, arfS, is an excellent candidate. However, for us, it is not ideal for the following reasons.

1. The xyzA, xyzB, xyzC-- format is intended to represent different genes or cistrons involved in a biological function in one organism. Thus, use of the A,

B, C part to indicate different organisms can be confusing. In this sense, arfA, and arfT are already confusing.

2. The *yqkK* homologs are present in some *Bacillus* genus, not only in *Bacillus subtilis*, making *arfS* not ideal. We cannot use "B" either, because ArfB is already used for *E. coli* ArfB.
3. As discussed in the paper, there is a possibility that *Bacillus subtilis* possesses yet unidentified ribosome rescue pathways. If a still new gene is discovered in the future, the naming of *arfS* will add another confusion.

After careful consideration of these and other issues, we decided to use a new nomenclature *brfA*/BrfA (*Bacillus* ribosome rescue factor A).

Reviewer #2 (Remarks to the Author):

The manuscript by Chiba and Wilson reported the discovery and mechanistic dissection of an alternative ribosome rescue factor in Gram-positive bacterial species. Overall, this is a very well executed study, with convincing data from bacterial genetics, biochemistry and structural biology. I recommend the publication of this manuscript after addressing the following issues.

Response: We appreciate the positive evaluation of our work.

While the majority of data analysis and interpretation is solid, a major concern is that the authors tended to claim ResQ being an independently evolved rescue factor. Since ResQ and ArfA are both small proteins, and their interactions with the ribosome (and RF2) are mostly not very much sequence-specific, it is highly likely they are actually products of divergent evolution. From a structural point of view, although detailed interactions between the factor and the ribosome (and between RF2 and the factor) are different in some regions for ArfA and ResQ, they display high similarities in many aspects: for example, the beta-strand interaction with RF2; two structurally conserved C-terminal residues (His and Lys); the ability to interact and change the switch region of RF2; and the binding of N-terminus in H69-H44 region. Therefore, it is hard to imagine that they do not have a common ancestor.

Response: We agree with the reviewer that we cannot rule out the possibility that ArfA and BrfA evolved from a common ancestor in divergent manners. However, without concrete evidence, we believe it is useful to propose an evolutionary scenario that we envisage being likely at this point. To make it clear that the discussion represents our hypothesis but not the established fact and to confirm to what the reviewer requests, we added a phrase "Although we do not rule out the possibility that BrfA and ArfA share the same evolutionary origin, (line 268)"

We also modified a sentence in Discussion by inserting the phrase "Assuming that BrfA and ArfA are evolutionarily unrelated. (line 521)"

A few minor issues:

1. Line 239 GAQ mutation of RF2 no longer stimulated the hydrolysis of GFP-tRNA in the presence of ResQ. Is there special consideration that GGP mutation, instead of GAQ was used to perform cryo-EM analysis (line299)?.

Response: The *B. subtilis* RF2 was mutated to GGP as it was previously shown to inactivate the catalytic activity of the release factor and was successfully crystallize in complex with *Thermus thermophilus* 70S ribosome and deacylated fMet tRNA (Santos et. al PMID: 23769667). The reason for using GGP instead of GAQ mutant is that we were hoping to have a better resolution structural feature of this region. Unfortunately, the resolution of the cryo-EM map in this region is very similar to the GAQ in the *E. coli* ArfA structure previously published.

2. U1915 mentioned in line 324 was not labeled in fig. 4f.

Response: U1915 is now labelled in Fig. 4f

3. There appear to be duplicate panels between Figure 4 and Supplementary Fig. 5, between Figure 5 and Supplementary Fig. 6, and between Figure 5 and supplementary Fig. 7.

Response: The figures were intentionally duplicated in the supplementary figures in order to have a better understanding of the overlay figures that are respectively displayed in the neighboring panels.

Reviewer #3 (Remarks to the Author):

In the manuscript by Shimokawa-Chiba and colleagues, the authors discover a new ribosome quality control pathway that serves as an essential backup to the trans-translation based ribosome quality control system. This new pathway, which uses ResQ (renamed from YqkK), is the first release factor (RF) dependent system described in a Gram-positive bacterium. First, the authors use a genome-wide selection strategy to identify genes that are essential in B. subtilis when the trans-translation system is inactive. They then verify the essentiality of ResQ in this context, using alternative knockdown and rescue strategies. The authors then carried out a range of molecular biology and biochemical experiments to establish the regulatory mechanism of ResQ expression, and how ResQ itself functions in an RF2-dependent manner. Additionally, the authors define the species-selectivity of the ResQ system, finding it depends on the species-specific RF2, but not the bacterial source of the ribosome.

Finally, the authors use cryo-EM to determine the molecular basis for how ResQ recruits RF2 to stalled ribosome nascent-chain complexes, to define when RecQ is likely to function, i.e. on truncated mRNAs. It's particularly striking how the ResQ system has evolved by convergent evolution to harbor many of the features of the E. coli ArfA ribosome quality control system, even though the molecular details di er in key respects. This includes similarities to how ArfA expression is regulated. Overall, this is a really interesting paper, and will be of wide interest to the microbiology community as well as the translation field.

The paper is overall well written. The following points should be addressed by the reviewers as minor revisions.

Response: We again appreciate the reviewer's positive evaluation.

1. Given the convergent evolution seen here, and the two kinds of regulatory mechanism for generating trans-translation sensitive expression of ResQ and ArfA, the authors

could shorten some of the other discussion points a little, and add a paragraph on how the termination mechanisms used to regulate ResQ and ArfA expression could be used to potentially identify other ribosome quality control pathways in bacteria that serve as backup to the trans-translation system. This could be a fruitful way to identify sequence-divergent proteins that serve the same role as ResQ/ArfA and that are regulated in a similar manner. I think this would broaden the interest of the paper.

Response: Thank you for the excellent proposal. As the reviewer pointed out, BrfA and ArfA are both natural substrates of the *trans*-translation pathway while they share no sequence similarity. However, ArfT is not a natural target of *trans*-translation. The ArfA mRNA was reported to be cleaved by RNasIII, and this cleavage also contributes to the *trans*-translation sensitive expression of ArfA. Thus, we are not sure whether *trans*-translation sensitivity is a common feature of alternative ribosome rescue factors, and whether transcription termination is the only mechanism producing a truncated mRNA. Therefore, we decided not to mention, at this point, the attractive approach that the reviewer kindly proposed.

2. In many of the figure panels, the labels are light and hard to see. For example, in Figure 4 with ~blue-green labels on the yellow background of the 30S subunit, and some of the other light labels. There are a few cases in Figures 4, 5, Supplementary Figure 2a-b, and Supplementary Figures 5-7 where it may be necessary to use black lines to connect darker labels to the structural feature, rather than using light labels.

Response: We addressed this point by applying a very thin contour around the light labels and black lines between label and feature in some cases

3. Line 219-220. Please provide a citation (58?) and/or methods for the neutral-pH SDS gels. These may not be familiar to most readers. Also provide this citation on line 657, if this is the protocol used. Otherwise, please add details of the protocol.

Response: Following the reviewer's suggestion, we added a citation of the SDS-PAGE (ref. 31, formerly ref. 58) to both Line 225 and line 667.

REVIEWERS' COMMENTS:

Reviewer #1 (Remarks to the Author):

The authors have satisfactorily addressed the few issues raised by the reviewers, including adding new experimental data that the hairpin in BrfA in fact arrests transcription.
